# Supplementary material for: Programming Nonlinear Interfacial Mechanics of Synthetic Cells: Lipid Geometry and DNA Nanostructures
Source: Small Sci. 2026 Jun 16;6(6):e70321. doi: 10.1002/smsc.70321 (PMC13274316; doi:10.1002/smsc.70321)
Supplement: Supplementary file 1 — Supplementary Material [file SMSC-6-e70321-s001.pdf]

Supporting Information for

Programming Nonlinear Interfacial Mechanics of Synthetic Cells: Lipid Geometry and DNA Nanostructures

Kazutoshi Masuda[1] and Miho Yanagisawa[1,2,3]

[1]Komaba Institute for Science, Graduate School of Arts and Sciences, The University of Tokyo, Komaba 3-8-1, Meguro, Tokyo 153-8902, Japan
[2]Department of Physics, Graduate School of Science, The University of Tokyo, Hongo 7-3-1, Bunkyo, Tokyo 113-0033, Japan
[3]Center for Complex Systems Biology, Universal Biology Institute, The University of Tokyo, Komaba 3-8-1, Meguro, Tokyo 153-8902, Japan

S1 Volume and surface area of the aspirated region

Equations 3 and 4 are derived from a purely geometrical analysis of the membrane segment aspirated into the micropipette. The volume V of the aspirated region is given by the volume of a spherical cap with radius R' and polar angle θ, from which the cylindrical volume corresponding to the pipette interior is subtracted:

$$V = 2\pi \int_0^{R'} r^2\,dr \int_0^{\theta} \sin\theta\,d\theta - \frac{1}{3}\pi R_p^2(R' - L) = \frac{2}{3}\pi R'^3(1-\cos\theta) - \frac{1}{3}\pi R_p^2(R' - L) \tag{S1}$$

Using the geometrical relations $R' = \frac{R_p(1+x^2)}{2x}$, $\quad L = xR_p$, the expression reduces to

$$V = \frac{1}{6}\pi R_p^3(3x + x^3). \tag{S2}$$

Similarly, the surface area A of the aspirated spherical segment is obtained as

$$A = 2\pi R'^2 \int_0^{\theta} \sin\theta\,d\theta = 2\pi R' L = \pi R_p^2(1+x^2). \tag{S3}$$

S2 Estimation of bending energy at the micropipette edge

The bending energy associated with the membrane at the micropipette edge (Figure 1c) is evaluated by estimating the surface area of the rotational surface around the x-axis. The edge surface area A_{edge} is given by

$$A_{\text{edge}} = \int_{-h\cos\theta_c/2}^{-h\cos\theta_o/2} y\sqrt{1+(y')^2}\,dx, \quad y = R_p - \sqrt{\left(\frac{h}{2}\right)^2 - x^2}. \tag{S4}$$

This leads to

$$A_{\text{edge}} = \pi h\left[R_p(\theta_o - \theta_c) + \frac{h}{2}(\cos\theta_o - \cos\theta_c)\right]. \tag{S5}$$

The corresponding bending energy at the edge is approximated as

$$E_{\text{edge}} \approx \frac{1}{2}kA_{\text{edge}}\left(\frac{1}{h/2} - \frac{1}{R_p} - \frac{2}{R_0}\right)^2 \approx \frac{1}{2}kA_{\text{edge}}\left(\frac{1}{h/2} - \frac{2}{R_0}\right)^2, \tag{S6}$$

which yields

$$E_{\text{edge}} = 2\pi k\frac{R_p}{h}\left(1 - \frac{h}{R_0}\right)^2\left[\sin^{-1}\left(\frac{2x}{1+x^2}\right) - \theta_c\right]. \tag{S7}$$

Because $h \ll R_p, R_0$, this contribution is significantly smaller than the bending energy of the aspirated spherical segment and can therefore be neglected.

S3 Conversion from $P_{\text{diff}}(x)$ to the measured pressure $P(x)$

To relate the effective pressure difference $P_{\text{diff}}(x)$ to the experimentally measured aspiration pressure $P(x)$, we consider additional contributions arising from interfacial tension and bending elasticity outside the pipette. The Helfrich bending energy of a spherical membrane with radius R is given by

$$E_{\text{bend}} = 4\pi R^2 \cdot 2k\left(\frac{1}{R} - \frac{1}{R_0}\right)^2. \tag{S8}$$

The bending-induced pressure difference across the membrane is obtained as

$$\Delta P_{\text{bend}} = \frac{dE_{\text{bend}}}{dV} = \frac{dE_{\text{bend}}/dR}{dV/dR} \approx -\frac{k}{R}\left(\frac{1}{R_0}\right)^2. \tag{S9}$$

Adding this bending contribution to the classical Young–Laplace pressure yields

$$P(x) = P_{\text{diff}}(x) - \left[\frac{2\gamma(x)}{R} + \frac{k}{R}\left(\frac{1}{R_0}\right)^2\right]. \tag{S10}$$

Such generalizations of the Young–Laplace equation incorporating bending elasticity have been reported in previous studies.

S4 Estimation of I_{mean} and I_{sh} by fluorescence intensity quantification

To estimate the mean intensity of Y-motifs within the entire droplet (I_{mean}) and the mean intensity of Y-motifs localized beneath the droplet surface (I_{sh}), we measured the fluorescence intensity over the whole droplet cross section, I_{cross}, and the intensity within the inner region of radius R_{in}, denoted by $I_{\mathrm{in,cross}}$. Using these two quantities, I_{mean} and I_{sh} were estimated by taking into account the three-dimensional spherical geometry of the droplets, as follows:

$$I_{\mathrm{sh}} = \frac{I_{\mathrm{cross}} \cdot R^2 - I_{\mathrm{in,cross}} \cdot R_{\mathrm{in}}^2}{R^2 - R_{\mathrm{in}}^2} \tag{S11}$$

$$I_{\mathrm{mean}} = I_{\mathrm{sh}}(1 - \frac{R_{\mathrm{in}}^3}{R^3}) + I_{\mathrm{in,cross}} \frac{R_{\mathrm{in}}^3}{R^3}. \tag{S12}$$

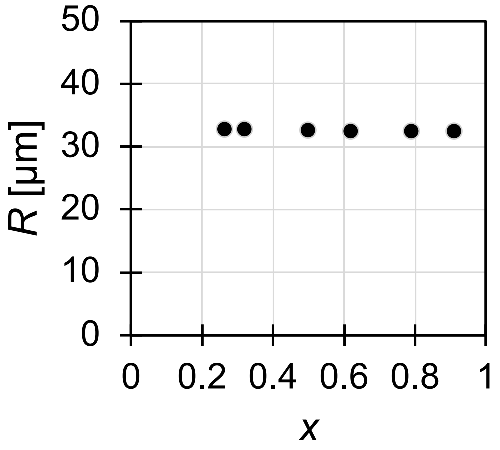

Figure S1: No change in the droplet radius R outside the pipette was observed during aspiration. The analysis was conducted with a TAP droplet encapsulating a 370 mM NaCl solution.

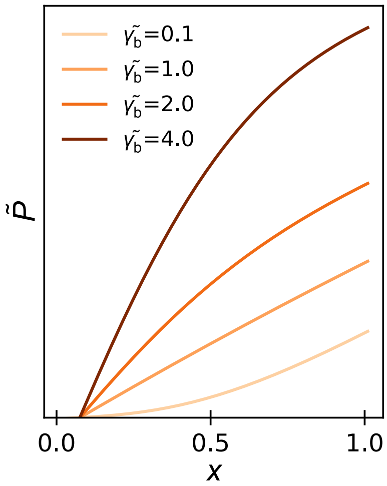

Figure S2: Plots showing the numerically obtained relationship between the normalized pressure $R_{\mathrm{p}}P/K \equiv P$ and the normalized aspiration length, x, for various values of $\gamma_0 + \widetilde{k} \equiv \widetilde{\gamma}_b$. The curve becomes convex upwards when $\widetilde{\gamma}_b$ is large and convex downwards when it is small.

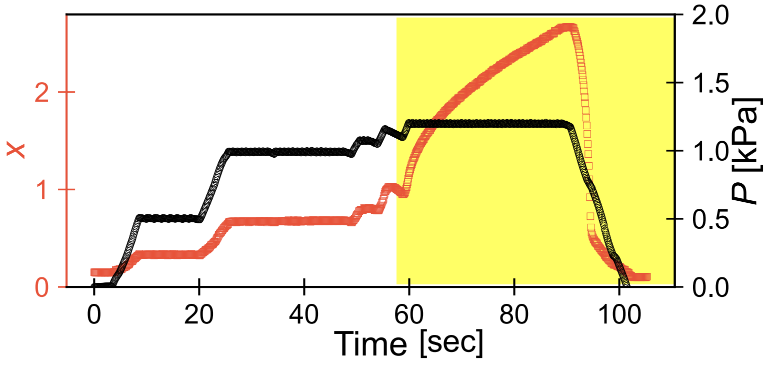

Figure S3: Change in x (red, left axis) as P (black, right axis) is gradually increased during the aspiration experiment. When $x \leq 1$, x remains nearly constant over time under a constant applied pressure P, indicating an elastic response. In contrast, when $x > 1$, x continues to increase with time even while P is kept constant (yellow-shaded region), indicating the onset of viscous behavior.

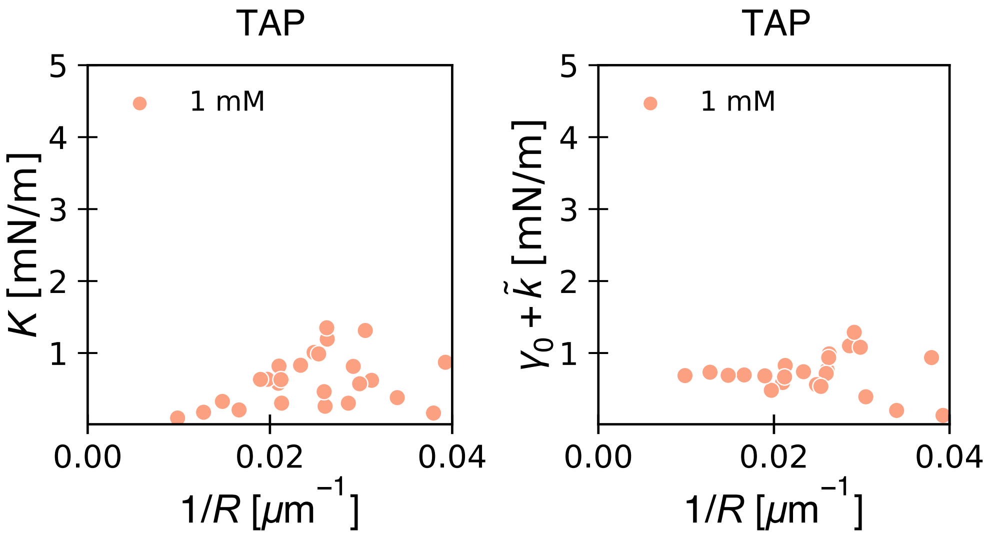

Figure S4: The curvature dependency of K (left) and $\gamma_0 + \tilde{k}$ (right) for TAP droplets.

G1: GCGCCAGTGAGGACGGAAGTTTGTCGTAGCATCGCACC
G2: GCGCCAACCACGCCTGTCCATTACTTCCGTCCTCACTG
G3: GCGCGGTGCGATGCTACGACTTTGGACAGGCGTGGTTG
S1: CAGTGAGGACGGAAGTTTGTCGTAGCATCGCACC
S2: CAACCACGCCTGTCCATTACTTCCGTCCTCACTG
S3: GGTGCGATGCTACGACTTTGGACAGGCGTGGTTG

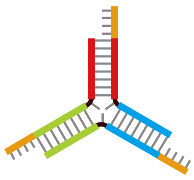

Figure S5: DNA sequences used to form the Y-motif. Sticky-end, which are marked as yellow, connect to the other sticky-end to make the network structure. Each color shows its complementary sequence.

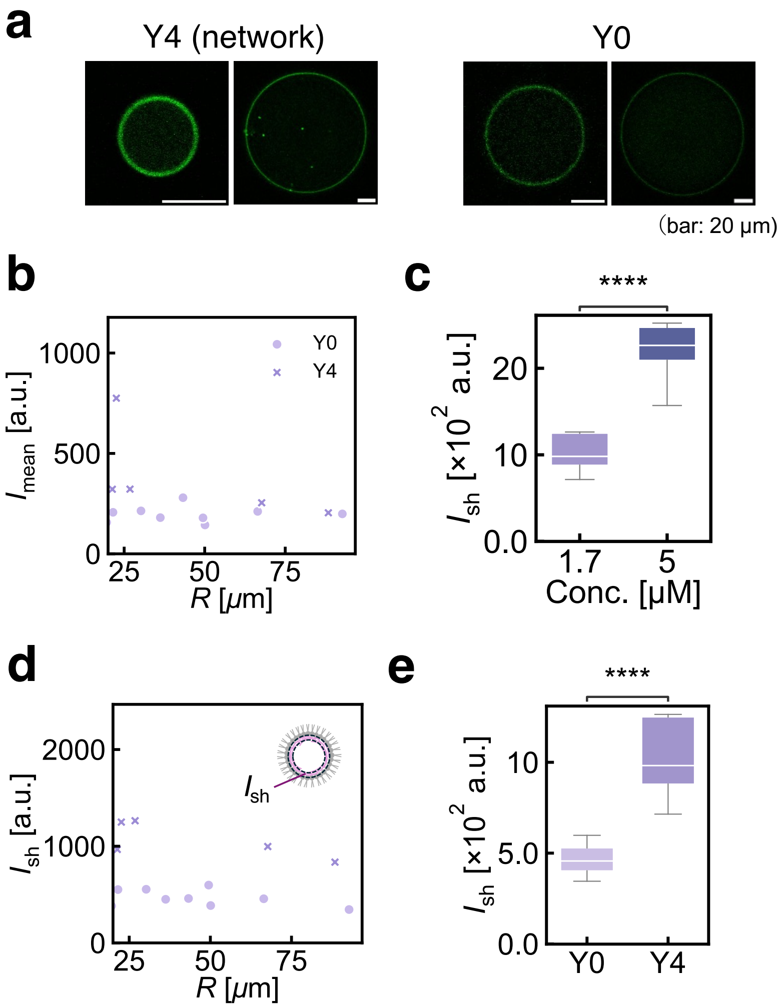

Figure S6: (a) Confocal cross-sectional images showing DNA nanostructures (green) inside synthetic cells: networked Y-motifs (Y4, left) and independent Y-motifs (Y0, right). (b) Relationship between the mean intensity of Y-motifs ($I_{\rm mean}$) and the radius (R) of synthetic cells. All samples show nearly identical $I_{\rm mean}$ values, indicating that the total amount of Y-motifs is essentially the same. (c) Comparison of the mean intensity of Y-motifs localized beneath the surface ($I_{\rm sh}$) between different concentrations of Y4 for data with $R \geq 20$ μm. (d) Relationship between $I_{\rm sh}$ and R. For each Y-motif, $I_{\rm sh}$ remains nearly constant when $R \geq 20$ μm. (e) Comparison of $I_{\rm sh}$ among different Y-motifs for data with $R \geq 20$ μm. Asterisks indicate statistical significance based on Welch's t-test, with **** indicating $p < 0.001$.

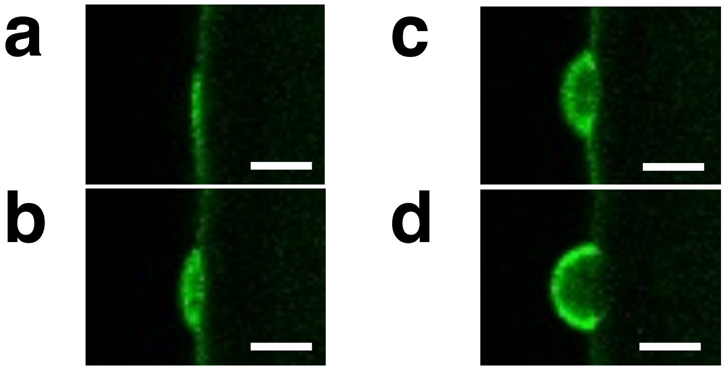

Figure S7: (a–d) Confocal cross-sectional images of droplets with DNA during micropipette aspiration. DNA nanostructures (green) remained uniformly distributed within the pipette. The apparent higher fluorescence intensity inside the pipette arises from the large membrane curvature, which leads to the accumulation of fluorescence signals from the upper and lower membranes. Scale bar is 20 μm.
